# Downregulated androgen receptor expression in preputial tissues of children with hypospadias

**DOI:** 10.3389/fped.2025.1660777

**Published:** 2025-08-29

**Authors:** Zhilin Yang, Peiliang Zhang, Tiejun Zhang, Jianchun Yin, Guanglun Zhou, Weiguang Zhao, Pengyu Chen, Jiaqiang Li, Shoulin Li

**Affiliations:** ^1^ Department of Urology and Laboratory of Pelvic Floor Muscle Function, Shenzhen Children’s Hospital, Shenzhen, Guangdong, China; ^2^ Department of Urology, Shenzhen Children's Hospital of Shantou University Medical College, Shenzhen, Guangdong, China

**Keywords:** hypospadias, androgen receptor, prepuce, children, expression

## Abstract

**Purpose:**

To resolve conflicting evidence on androgen receptor (AR) expression in hypospadias, we compared preputial AR levels between affected children and controls.

**Methods:**

Forty patients with isolated hypospadias (age 2.9 ± 0.79 years, range 1.6–5.2 years) and 40 normal boys (age 3.2 ± 0.79 years, range 1.8–4.7 years) who underwent circumcision for phimosis were included in our study. Inner plates of preputial tissues were analyzed by immunohistochemistry for AR detection.

**Results:**

AR immunoreactivity was predominantly localized to the basal epithelial layer. Significantly reduced AR expression was observed in hypospadias patients vs. controls [average optical density (AOD), 0.38 ± 0.09 vs. 0.56 ± 0.11]. However, there was no difference in AR expression between the proximal and distal hypospadias (AOD, 0.40 ± 0.07 vs. 0.37 ± 0.10, *P* = 0.22).

**Conclusions:**

Preputial AR downregulation in hypospadias implicates AR deficiency in pathogenesis, **providing evidence to resolve** a key controversy in the field.

## Introduction

Hypospadias is a common congenital malformation of the urinary system in male children, with a prevalence of 18.6 per 10,000 births (1). It is defined as incomplete development of the male urethra leading to aberrant localization of the urinary meatus on the ventral side of the penis in a variable position from the glans to the perineum. The etiology of hypospadias remains unknown. Although some studies have implicated androgen receptors (ARs) in hypospadias development, recent studies report conflicting results regarding AR expression.

Vottero et al. demonstrated that AR expression in the preputial tissue of patients with hypospadias was lower than that of normal children (2). Chao et al. examined the AR in the prepuce of patients with hypospadias and controls using immunohistochemistry (IHC) and observed that AR expression in 68 hypospadias tissues was significantly lower than that in 68 normal Preputial tissues (3). Silva T et al. studied 17 hypospadias and 41 control patients and observed significantly less AR mRNA levels in the urethral mucosa of patients with hypospadias than in controls (4). Recent epigenetic studies further highlight transcriptional dysregulation of AR in preputial mucosa (5, 6). In contrast, Pichler et al. discovered that AR protein was higher in boys with hypospadias than in boys without hypospadias (133.25 ± 6.17 vs. 100 ± 4.45, *P* = 0.014) (7). Similarly, Kocaturk et al. documented higher AR expression in hypospadias-affected preputial tissue than in normal tissue (8). Hence, the expression of AR in patients with hypospadias remains controversial.

We conducted a large prospective study to clarify discrepancies in AR expression between hypospadias patients and normal children, and to assess the relationship between AR dysregulation and hypospadias development.

## Materials and methods

### Patients and study design

From January 2022 to December 2024, 40 patients with isolated hypospadias and 40 healthy boys who underwent circumcision for phimosis were included in our study. All subjects were ethnic Han people of Chinese origin. Distal and proximal hypospadias were present in 24 and 16 patients, respectively. Patients with DSD, cryptorchidism, or other anomalies and those with a history of preoperative hormonal treatment were excluded. Preoperative blood samples were collected for hormone analysis; preputial tissues were harvested intraoperatively for histopathological examination.

### Hormone measurements

Serum dihydrotestosterone (DHT) levels were tested in all subjects before surgery. Blood samples were centrifuged immediately after collection. Then, the plasma was stored at −20 ℃ until assay. Serum testosterone and DHT levels were measured using commercial kits (ELISA, Mlbio Company, Shanghai, China). The sensitivity of the hormone assay was 99%.

### Immunohistochemistry (IHC)

The inner plates (mucosal surface) of prepuce tissues were procured intraoperatively during circumcision or hypospadias repair, fixed in 4% paraformaldehyde for ≥6 h, rinsed three times, and dehydrated through a graded series of isopropanol and xylene. Subsequently, tissues were embedded in paraffin, and 4 μm sections cut at 200 μm intervals were prepared for staining.

For AR detection, sections underwent standardized immunohistochemistry:

• Deparaffinization in xylene;
• Heat-induced antigen retrieval (100°C, 20 min in citrate buffer, pH 6.0);
• Primary antibody incubation with monoclonal mouse anti-human AR antibody (AR441, MXB Biotechnologies; 1:100 dilution, 8 h at 4°C);
• Secondary antibody incubation with biotinylated goat anti-mouse IgG (37°C, 2 h);
• Signal amplification with streptavidin-HRP (30 min);
• DAB chromogenic development (monitored in real-time under microscopy);
• Counterstaining with hematoxylin (1 min).

All procedures were conducted by a single blinded pathologist.

Human prostate carcinoma tissue served as the positive control. AR immunoreactivity was quantified as average optical density (AOD) using ImageJ (NIH v1.53k). For each specimen, five random fields per section (totaling 25 fields/sample) were analyzed via superimposing a 100-point grid. Mean AOD values were compared among hypospadias patients, controls, and distal/proximal subtypes.

### Institutional review board approval

This study was approved by the Institutional Review Board of Shenzhen Children's Hospital (IRB No. 202210802), and informed consent was obtained from the parents of the children.

### Statistical analyses

Continuous data were compared using independent t-test. Categorical variables were analyzed with the chi-squared test or logistic regression analysis. All statistical analyses were performed with a two-tailed significance level of *α* = 0.05 using SPSS 26.0 (IBM Corp., Armonk, NY).

## Results

The hypospadias group included 40 patients; the mean age was 2.90 ± 0.79 (range, 1.6–5.2) years. Forty patients who underwent circumcision were in the control group, and the mean age was 3.2 ± 0.79 (range: 1.8–4.7) years. There was no difference in age between the hypospadias and control groups (Table 1). In addition, there was no difference in serum DHT concentration between the hypospadias and control groups (0.38 ± 0.11 vs. 0.39 ± 0.13 nmol/L, *P* = 0.88).

**Table 1:** AR expression in the inner plate of prepuce in hypospadias vs. control groups.

Parameter	Hypospadias group	Control group	*P*-value
Number	40	40	
Age (year)	2.90 ± 0.79	3.20 ± 0.79	0.11
AOD of AR	0.38 ± 0.09	0.56 ± 0.11	0.01
DHT (nmol/L)	0.38 ± 0.11	0.39 ± 0.13	0.88
Associated malformations	0%	0%	>0.05

AOD, average optical density; AR, androgen receptor; DHT, dihydrotestosterone.

**Figure 1:**
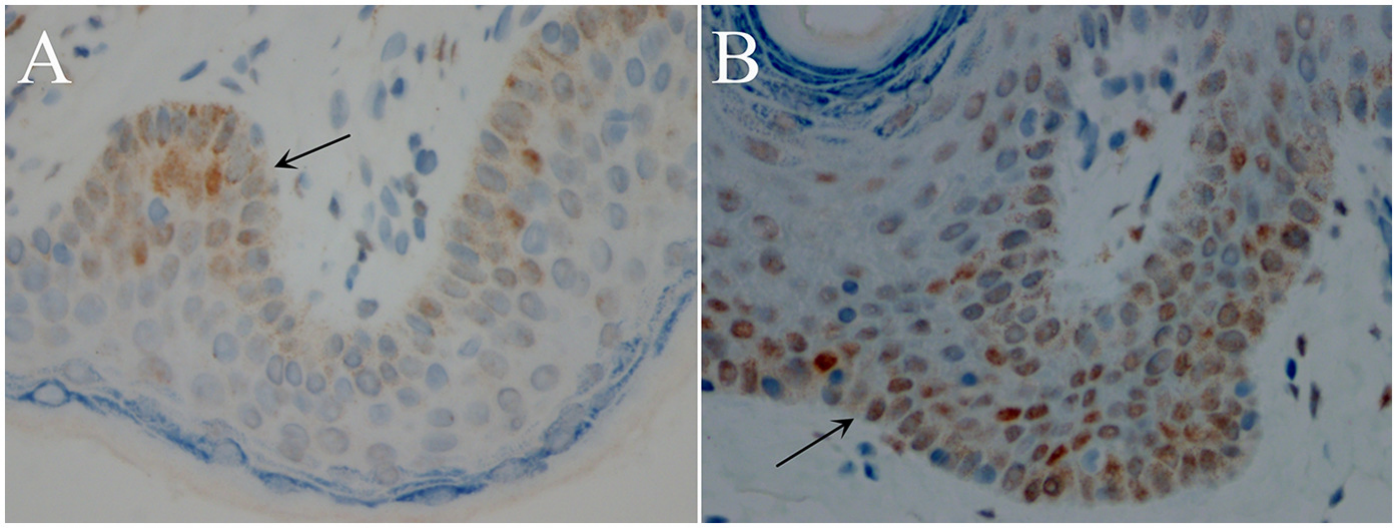
Androgen receptor (AR) immunostaining in inner plate preputial tissues (400×, transverse sections). Strongest nuclear staining in basal epithelial layer (arrows). **(A)** Hypospadias group: Sparse immunopositive cells with weak staining intensity. **(B)** Control group: Numerous strongly stained immunopositive cells. DAB chromogen, hematoxylin counterstain.

IHC of inner plate preputial tissues revealed that the AR protein was majorly expressed on the basal cell layer, while some were expressed in the fibroblasts (Figure 1). Furthermore, AR expression was lower in the hypospadias group than in the control group (AOD, 0.38 ± 0.09 vs. 0.56 ± 0.11, *P* = 0.01) (Table 1) (Figure 1). However, there was no difference in AR expression between the proximal and distal hypospadias (AOD, 0.40 ± 0.07 vs. 0.37 ± 0.10, *P* = 0.22) (Table 2).

**Table 2:** AR expression in the inner plate of prepuce in distal vs. proximal hypospadias.

Parameter	Distal hypospadias group	Proximal hypospadias group	*P*-value
Number	24	16	
Age (year)	2.87 ± 0.89	2.96 ± 0.64	0.75
AOD of AR	0.40 ± 0.07	0.37 ± 0.10	0.22
DHT (nmol/L)	0.38 ± 0.10	0.38 ± 0.11	0.96
Associated malformations	0%	0%	>0.05

AOD, average optical density; AR, androgen receptor; DHT, dihydrotestosterone.

## Discussion

Our study demonstrated significantly reduced AR expression in hypospadias patients compared to healthy controls, with no differential expression between proximal and distal subtypes.

Although extensive studies have characterized the histology of hypospadias, its etiology remains elusive. Multiple genes—including ZEB1, Hsa_circ_0000417, SRY, 5α-reductase, and AR—have been implicated as contributors, though none are established as causative (2, 3, 9).

The second trimester is a critical period for human fetal urethral development. During this period, androgens are critical for the differentiation and development of sexual genitalia in male fetuses (11). DHT-AR signaling is essential for urethral closure, with AR functioning as a ligand-dependent nuclear receptor transcription factor (12). AR deficiency disrupts androgen signaling, impairing male sex differentiation and potentially contributing to hypospadias pathogenesis.

The predominant localization of AR to the basal epithelial layer is biologically significant, as this compartment contains progenitor cells critical for urethral morphogenesis. Basal AR signaling likely orchestrates epithelial-mesenchymal interactions (EMT) during urethral closure by regulating downstream targets (e.g., ZEB1, FGF10). Reduced AR in basal cells may disrupt EMT, impairing urethral tubularization, resulting in failed urethral fusion and contributing to hypospadias pathogenesis (9, 10).

Numerous studies have investigated the AR-hypospadias relationship, yet yielded conflicting results. Vottero et al. reported reduced AR expression in preputial tissue from hypospadias patients vs. healthy controls (2). Conversely, Pichler et al. documented elevated AR protein in affected boys (133.25 ± 6.17 vs. 100 ± 4.45, *P* = 0.014) (7). These discrepancies likely reflect methodological variations and limited sample sizes in prior studies. To resolve this controversy, we employed a large cohort to definitively establish the AR-hypospadias association.

Our study demonstrated that AR expression is significantly decreased in patients with hypospadias than in healthy boys. This may be associated with the etiology of hypospadias. Lower AR expression causes abnormal combination and dysfunction of androgens; thus, urethral development is affected, and urethral closure cannot be achieved, contributing to various hypospadias. Vottero et al. documented reduced AR in preputial tissue from affected patients vs. controls (2), a result corroborated by Chao et al. using immunohistochemistry (3). Silva et al. further reported diminished AR mRNA in urethral mucosa (4), while An et al. noted AR reduction in penile skin, particularly within the urethral plate (10). Consistent with our protein-level findings, Yıldız et al. demonstrated downregulated AR expression and associated DNA methylation changes in foreskin tissue of hypospadias patients (6). Similarly, İnanç et al. reported dysregulation of AR and related genes (ESR1, FGFR2) in preputial tissues, reinforcing end-organ resistance in hypospadias pathogenesis (5). This convergence of protein and gene evidence reinforces AR deficiency as a hallmark of hypospadias. Conversely, several groups report contradictory data. Kocaturk H. described increased AR expression in affected preputial tissue (8). Balaji et al. similarly noted heightened AR levels, with proximal subtypes exhibiting greater expression than distal forms, potentially indicating end-organ overexpression (13).

We observed no difference in AR expression between the proximal and distal hypospadias. Although AR mediates external genitalia development, its levels did not correlate with phenotypic severity—a finding consistent with Celayir et al. who reported no AR-severity association (14). In contrast, Qiao et al. described elevated AR in severe vs. mild hypospadias (*P* < 0.05), though small sample sizes preclude definitive conclusions (15). Our null finding regarding AR expression across proximal/distal subtypes suggests that baseline AR deficiency may be a universal feature in hypospadias irrespective of severity. We speculate that compensatory mechanisms (e.g., local androgen synthesis) or alternative signaling pathways may modulate phenotypic expression beyond AR abundance alone.

Impaired AR transcriptional activity in hypospadias may be ameliorated by androgen supplementation. Kaya et al. demonstrated that preoperative DHT gel reduces postoperative complications (e.g., fistula rates) and improves cosmetic outcomes, potentially via enhanced angiogenesis and signaling rescue (16). Our data support DHT trials in AR-deficient hypospadias, reinforcing this therapeutic approach.

Our findings contrast with prior reports of AR overexpression in hypospadias (7, 8, 13). Specifically, we observed reduced AR expression in patients vs. healthy controls. These discrepancies likely reflect methodological differences. Pichler et al. quantified AR in whole-tissue lysates via Western blotting, whereas our IHC precisely localized AR to basal epithelial cells—the primary site for androgen signaling during urethrogenesis (7).

While our study identifies AR downregulation, future work should explore upstream mechanisms (e.g., AR promoter methylation, miRNA regulation) and functional consequences via *in vitro* models of urethral development.

In conclusion, the inner plate preputial tissues of patients with hypospadias showed decreased AR expression, suggesting that AR may be involved in hypospadias development and contributes to resolving a key controversy. However, further studies are necessary to assess whether epigenetic abnormalities or gene mutations are responsible for the decreased AR expression.

## Data Availability

The original contributions presented in the study are included in the article/Supplementary Material, further inquiries can be directed to the corresponding author.
